# RootGraph: a graphic optimization tool for automated image analysis of plant roots

**DOI:** 10.1093/jxb/erv359

**Published:** 2015-07-29

**Authors:** Jinhai Cai, Zhanghui Zeng, Jason N. Connor, Chun Yuan Huang, Vanessa Melino, Pankaj Kumar, Stanley J. Miklavcic

**Affiliations:** ^1^Phenomics and Bioinformatics Research Centre, University of South Australia, Mawson Lakes SA 5095, Australia; ^2^Australian Centre for Plant Functional Genomics, University of Adelaide, Hartley Grove, Urrbrae SA 5064, Australia

**Keywords:** 2D, fully automated, graphic optimization, high throughput, image analysis, root network analysis, root phenotyping, wheat and barley.

## Abstract

This paper outlines a numerical scheme for accurate, detailed, and high-throughput image analysis of plant roots. In contrast to existing root image analysis tools that focus on root system-average traits, a novel, fully automated and robust approach for the detailed characterization of root traits, based on a graph optimization process is presented. The scheme, firstly, distinguishes primary roots from lateral roots and, secondly, quantifies a broad spectrum of root traits for each identified primary and lateral root. Thirdly, it associates lateral roots and their properties with the specific primary root from which the laterals emerge. The performance of this approach was evaluated through comparisons with other automated and semi-automated software solutions as well as against results based on manual measurements. The comparisons and subsequent application of the algorithm to an array of experimental data demonstrate that this method outperforms existing methods in terms of accuracy, robustness, and the ability to process root images under high-throughput conditions.

## Highlight

The method presented analyses root scans automatically, distinguishes primary from lateral roots, and quantifies a broad range of traits for individual primary roots and their associated lateral roots.

## Introduction

Plant root development, root systems and their 3D architecture (RSA) have been subjects of extensive study for many decades (Gregory *et al.*, 1987). This interest derives from the fact that roots are critical for plant stabilization and are the principal organs responsible for the uptake of water and nutrients from the soil. It is well known that RSA is under genetic control but can be significantly influenced by environmental factors [the genotype -by- environment (G×E) interaction] (Fitter, 2002; Masle, 2002); the phenotype corresponding to a specific genotype is environmentally dependent (Crossa, 2012; El-Soda *et al.*, 2014). Specific studies have documented the effects of abiotic stress on plant growth and adaptation and identified quantitative trait loci (QTLs) associated with important root traits (Kamoshita *et al.*, 2008). With this achieved, the screening of large mapping populations then calls for high-throughput analysis methods to deal with the large number of samples possible through root plasticity (Ardiel *et al.*, 2002; Kamoshita *et al.*, 2008). Specifically, an important, if not indispensable, component in any high-throughput phenotyping pipeline is a robust, accurate and fully automated image analysis tool.

Despite the importance to plant phenotyping (Gerlai, 2002; Niklas and Enquist, 2002; Furbank, 2009; Harris *et al.*, 2010), the development of automated and high-throughput analysis tools is in its adolescence, while root-based analysis specifically is in its infancy. In the past, root phenotypes were largely determined by means of expert human observations on detached roots. Biologists relied on visual inspection to draw comparisons between different root systems and relied on linear rulers to measure the lengths of plant primary roots (Kramer and Boyer, 1995; Sabatini *et al.*, 2003). However, to gain a full understanding of the G×E interaction and particularly the plant response to abiotic stresses, more detailed and more complete quantitative analyses are required.

Non-destructive, image-based techniques and image analysis algorithms have been developed to reconstruct in real time, the root system architecture of plants grown in gel-based platforms (French *et al.*, 2009; Clark *et al.*, 2011; Lobet *et al.*, 2011; Diener *et al.*, 2013; Kumar *et al.*, 2013). However, an inherent disadvantage of these systems is their limited representation of actual root development of plants grown in soils; the physical and chemical differences between soils and gel-based media result in distinctively different plant growth behaviour. In order to observe plant roots grown in soils, X-ray micro-tomography has been adopted for root phenotyping (Gregory *et al.*, 2003). As with its counterpart in the area of medical imaging, this level of technology although desirable, is costly to establish as well as to use. As a compromise measure, plants can be grown in thin rhizotrons—soil-filled cavities between parallel glass or transparent PVC slides (Price *et al.*, 2002; Leitner *et al.*, 2014). This gives rise to pseudo 3D systems that allow for root growth and development to be observed and imaged, at least as relates to the part of the root system that comes into contact with the transparent walls. The analysis of these 2D root images can thus provide information about some root traits for these pseudo 3D, soil-grown plants.

The most common approach to characterize roots grown in soils is to measure root traits after root extraction and washing (Iyer *et al.*, 2010; Pierret *et al.*, 2013). Although excavation and soil core washing in this approach destroys the 3D topology of the root system, it has been extensively used in phenotypic analyses. Indeed, even without knowledge of 3D architecture, it provides significant information on root numbers, root length, and root volume (Dowdy *et al.*, 1998; Pierret *et al.*, 2013; Kumar *et al.*, 2014). Although the preparation procedure itself (root extraction, washing, cutting, and spreading on a flatbed scanner) is a tedious bottleneck for high-throughput phenotyping, the manual task of analysing scans is an additional bottleneck. Moreover, manual analysis is the component that is most prone to subjective errors.

Image analysis techniques have been widely adopted for fast and reliable root phenotyping and made available through commercial software solutions such as WinRHIZO^TM^ and open source software such as EZ-Rhizo (Armengaud *et al.*, 2009), IJ_Rhizo (Pierret *et al.*, 2013), SmartRoot (Lobet *et al.*, 2011), RootTrace (Clark *et al.*, 2013), RootNav (Pound *et al.*, 2013), and Root System Analyzer (Leitner *et al.*, 2014). With the assistance of image analysis it becomes possible to determine quantitative features such as root numbers, root diameters, root lengths as well as diameter and length distributions. These semi and fully automated software solutions were designed to analyse roots of seedlings displayed in high quality 2D scans. For adult plants, root systems can be extensive and very complex, which also exacerbates the difficulty of physically removing soil from roots. Adult root systems are thus particularly challenging to analyse, and even more so when a significant level of noise is present in root scans due to remnant soil particulates attached to root hairs. For these basic reasons, existing tools such as WinRHIZO^TM^ and EZ-Rhizo, may not generate accurate root properties, such as root-tip counts (Kumar *et al.*, 2014). To accurately determine root number and root length, etc. it is necessary for any image analysis method to be robust to noise.

As another consideration, the identification of primary roots and lateral roots as distinct objects is also important to quantify tissue- as well as organ-specific responses to abiotic and nutrient deficiency stresses. For instance, a localized supply of nitrate, phosphate, or ammonium stimulates lateral root development in barley, while the primary roots are unresponsive (Drew *et al.*, 1973; Drew, 1975). For overall root phenotyping purposes, it is essential to quantify the development of primary roots and lateral roots over time as well as the relationship between lateral roots and primary roots. Currently, several software tools such as SmartRoot (Lobet *et al.*, 2011), ARIA (Pace *et al.*, 2014), RootNav (Pound *et al.*, 2013), and Root System Analyzer (Leitner *et al.*, 2014), and the authors’ recent software (Kumar *et al.*, 2014), referred to hereafter as *RTipC*, can separate primary roots from lateral roots. However, apart from RTipC, these tools are only semi-automated and so are not optimal for use in a high-throughput root phenotyping pipeline.

For the specific purpose of 2D root image analysis, one can consider the most common methods available, which are listed in Table 1 along with summaries of their capabilities. It is clear that a number of these can provide estimates of useful root characteristics from images in a semi-automatic way. However, there appears to be no tool that can also separate information about primary roots from that of lateral roots in a fully automatic way. Only a few of the available tools are designed to estimate the root (tip) number per primary root or per plant, which is one of the most important traits for root phenotyping (Armengaud *et al.*, 2009). Of these, ARIA (Pace *et al.*, 2014) requires the least user involvement. Furthermore, most use a skeletonization method to generate root representation and to count root tips by counting endpoints of the root skeleton. Regrettably, the traditional skeletonization method is sensitive to noise, thus these methods are not optimized for applications to soil-grown plants, whose scanned images may contain a significant number of soil particles contributing to noise. In summary, there is scope for further improvement. Indeed, from the user perspective there is a need for an analysis method that can automatically extract quantitative information about a root system and specifically distinguish between information related to primary roots from information on lateral roots, and do so from root images containing a relatively high level of noise.

**Table 1.** Summary of currently available root image analysis tools and their respective basic capabilities

Software	Automation	Topology	Root identification	Root count	Root diameter	Time series	RSML support Lobet *et al.* (2015)	Reference
ARIA	Automated	Yes	Yes^*a*^	No	No	Yes	No	Pace *et al.*, 2014
EZ-RHIZO	Automated	Yes	Yes^*b*^	No	No	No	No	Armengaud *et al.* (2009)
RootNav	Semi-Auto	Yes	Yes^*c*^	Yes^*d*^	No	No	Yes	Pound *et al.* (2013)
RootReader2D	Automated	Yes	Yes^*e*^	No	No	No	No	Clark *et al.* (2013)
RootSystemAnalyzer	Automated	Yes	Yes^*f*^	No	Yes	Yes	Yes	Leitner *et al.* (2014)
RootTrace	Automated	Yes	Yes^*c*^	No	No	Yes	No	French *et al.* (2009)
SmartRoot	Semi-Auto	Yes	Yes^*c*^	Yes^*c*^	Yes	Yes	Yes	Lobet *et al.* (2011)
RTipC	Automated	No	Yes^*g*^	Yes	No	No	No	Kumar *et al.* (2014)
WinRHIZO	Automated	Yes	Yes^*h*^	Yes	Yes	No	No	
RootGraph	Automated	No^i^	Yes	Yes	Yes	No	No	This work

^*a*^ Requires manual selection of source points.

^*b*^ Requires manual confirmation but users cannot correct errors.

^*c*^ Requires manual labelling of root types.

^*d*^ Detects only few root tips.

^*e*^ Labels roots by GUI in an interactive way.

^*f*^ Primary roots need manual initialization.

^*g*^ Distinguishes primary roots from lateral roots but does not separate the whole root system.

^*h*^ Based on a manual threshold of root diameter.

^*i*^ This is possible (as with RootNav) but not a current feature of this work.

In this paper, a robust approach to fully automated root analysis is provided using a combination of image processing and graphics optimization techniques. The approach, which is penned *RootGraph*, is robust to noise caused by soil particles or sand attached to roots hairs. As a result, it is able to accurately estimate the number, length, and diameter of roots. Moreover, the method is designed to automatically separate primary roots from lateral roots. Consequently, it can quantify traits for each primary root as well as each primary root’s associated lateral roots, as does SmartRoot, but can do so under high-throughput conditions. The accuracy of the algorithm is demonstrated through a performance comparison with alternate methods applied to cereal plant roots. The results confirm the robust and accurate performance of the method and demonstrate its capability for high-throughput root trait analyses. Although high performance is demonstrated through application to wheat and barley images, both monocots, the method is equally applicable to scanned images of dicots.

**Fig. 1.**
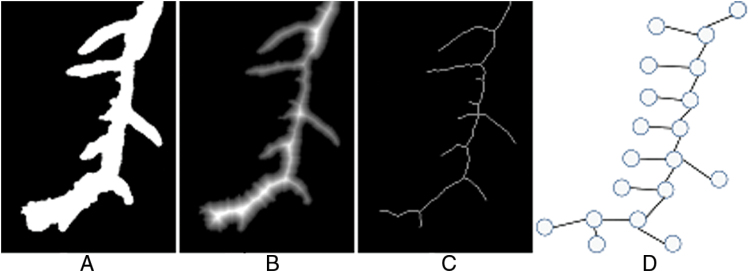
Illustrative examples of the four-layer data structure utilized in the new method: (A) the segmented image; (B) the Distance Transform of (A); (C) the skeleton of (A); and (D) the generated graph from the skeleton.

**Fig. 2.**
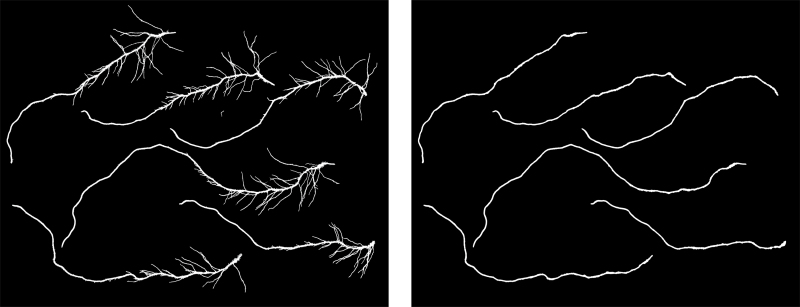
Illustrative example of the result of application of RootGraph to a flatbed scanned image of roots. The algorithm specifically identifies and separates primary from lateral roots. Left: original segmented root image; Right: extracted primary root image.

As a brief, non-technical description, the essence of the presented approach is based on a four stage program involving four layers of data structure. These layers comprise: segmented images, Distance Transform images, root skeleton images, and computer graphs. Case examples of each are shown in Fig. 1. Image segmentation distinguishes background from the plant root foreground. The Distance Transform allows adjustment of image resolution for subsequent processing, while root skeleton images allows the automatic creation of graphs to represent roots. The reference to the name RootGraph is in recognition of the use of graphs and of a graph optimization algorithm to automatically separate lateral roots from primary roots, as demonstrated by Fig. 2, and to analyse root traits accordingly. A few other approaches such as RootNav (Pound *et al.*, 2013) and ARIA (Pace *et al.*, 2014) also use graphs to represent roots and specifically also use the A* algorithm to find shortest paths. However, such direct minimization procedures may not lead to correct root characterization when root overlap is common. Root overlap is a recognized problem introduced during the preparation of scanned images of complex root systems. The problem is solved manually by RootSystemAnalyzer (Leitner *et al.*, 2014). In the method presented here, graph structure is used to represent the underlying root system, but a score or weight is assigned to each root segment to help distinguish primary from lateral roots. The optimization procedure is then applied to identify primary roots by maximizing the overall score. It is believed that RootGraph is the first method to employ a weighted graph-based optimization step to produce a fully automated, primary root identification procedure.

The method is elaborated on in the next section, with further information in the Appendix. Also in the next section, details of the experimental systems to which the RootGraph algorithm has been applied are provided.

## Materials and methods

### Plant material

Seeds of Australian barley (*Hordeum vulgare*) and wheat (*Triticum aestivum*) cultivars Kukri and Gladius were germinated in pots (17.5×8.5×8.5cm) filled with a potting mix of 50:50 Waikerie sand: coco-peat prepared with lime, gypsum, superphosphate, iron sulphate, iron chelate, potash (K_2_SO_4_), micromax (Osmocote) and pH adjusted to pH 6. Urea (56mg N/ kg soil) was added to the soil used to grow all barley plants as well as the wheat plants grown in the normal nitrogen (NN) treatment. The wheat plants grown in low nitrogen treatment (LN) were not supplied with urea but the soil contained residual nitrogen (15mg N/ kg soil).

Plants were grown in a controlled-environment growth room with 12h light/12h night cycle at 300 μmol m^−2^ s^−1^ photon flux intensity at the plant level, and temperature of 15 °C day /10 °C night for 14 d (barley) or 6, 9, 13 (Kukri wheat cultivar), and 17 d (Gladius wheat cultivar). Roots were washed free of soil particles and debris, cut from the root-shoot junction, and kept in an 30% ethanol solution until scanning. Before scanning, roots were spread out in a root positioning tray (20×30cm) to minimize overlap and scanned with a flatbed scanner (EPSON, EU-88, Japan). Greyscale images obtained in the tiff format were analysed with WinRHIZO^TM^ (Pro Version 2005a; Regent Instruments Inc., Canada). The settings used were as follows: image resolution, 600 dpi; calibration, intrinsic for the scanner; manual - dark root on white background; architecture by fractals: maximal pixel size (2.0mm) and filters, a length:width ratio smaller than 2.00. Root diameter (> 0.338mm) was set for primary roots and ≤ 0.338mm for lateral roots.

Roots were placed in a 20×30cm tray with 1cm of water and scanned on a flatbed scanner (dimensions) at 800 or 600 dpi.

### RootGraph: root image analysis with graph optimization

#### Segmentation

As a working principle, software tools should be designed with the ability to analyse root images obtained using different imaging tools such as flatbed scanners, and with these possibly set at different resolutions. Optimally, software solutions should thus be able to automatically adapt to variations in root images. The method employed here for segmenting plant root images has been described in detailed by Cai and Miklavcic (2013). The aim of segmentation is to separate foreground (plant roots) from background in such a way that the boundary between background and foreground can be determined easily by an edge detection method. Alternately, the edge detection method can be used to detect the boundary between foreground and background as well as to classify background points and foreground points. However, while all boundary points are edge points not all edge points are boundary points. To resolve the ensuing problem, surface fitting is used with the RANSAC algorithm to produce an accurate estimate of surfaces and thus segment plant roots from background. The major advantage of this approach is that the background of a plant root image does not have to be homogeneous and no manual threshold is needed.

#### Initial estimation of root diameter

**Fig. 3.**
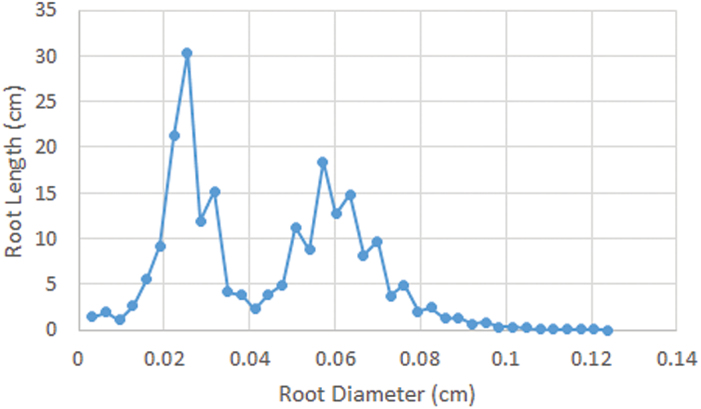
A typical root length vs root diameter histogram derived from a single root scan. Note the two distribution peaks attributed to primary and lateral roots. A colour version of this figure is available at *JXB* online.

In this case, the algorithm is required to estimate accurately root thickness or diameter at any point along the root as well as the average thickness of primary roots and of lateral roots. The Distance Transform was used to calculate the perpendicular distance from boundaries, as detected in Section B.1, to medial axis points along the root. With the Distance Transform, the root thickness at every medial axis point can be estimated. However, it is difficult to estimate the average thickness of lateral roots and primary roots without first establishing to which class a given root belongs. Here, the Gaussian mixture models (GMMs) and the histogram of root thicknesses was used, as shown in Fig. 3, to estimate the average thickness of lateral roots and primary roots. Let *H*(χ) be the histogram value of root thickness, χ, and let *P*(χ) be the density function defined as follows

$$P\left(\chi \right)=\sum_{\;k=1}^{\;K}W_{k}N\left(\chi |\mu_{k},\sigma_{k}\right)$$ (1)

where μ_k_, σ_k_, and *W*_k_ are the mean, standard deviation, and weighting, respectively, for the *k*th distribution in the above sum; *K* is the total number of distributions in the mixture, and

N(χ|μk,σk)=1σk2πexp(−(χ−μk)22σk2) (2)

is the single state Gaussian distribution. The parameters μ_k_, σ_k_, and *W*_k_ can be estimated using the Expectation–Maximization algorithm (Dempster *et al.*, 1977) to minimize the mean squared error of $H(\chi )/N_{m}-P(\chi )$, where *N*_m_ is the number of medial axis points. In this application, two types of roots are dealt with, therefore *K*=2. In this way, the average thickness of primary roots and lateral roots can be estimated.

#### Root thinning

**Fig. 4.**
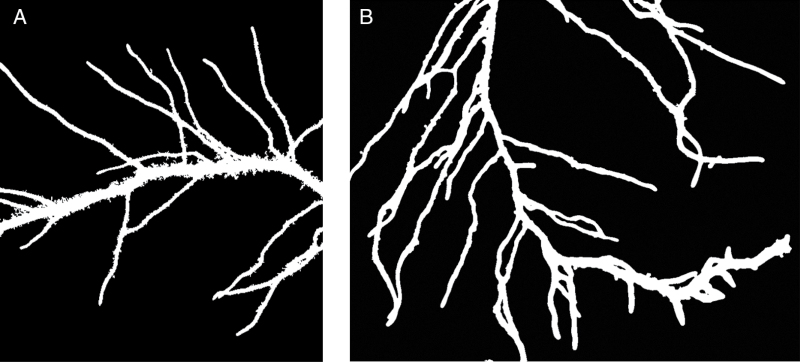
Segmented images of scanned roots demonstrating different levels of noise associated with the presence of remnant soil particles. (A) noisy roots; and (B) relatively clean roots. Note in (B), however, the occurrence of root overlap (left centre edge and bottom right corner), which requires the operation of a particular step in the graph analysis procedure to avoid incorrect root length estimation.

The traditional thinning algorithm aimed at reducing an image feature to a skeleton was first described by Zhang and Suen (1984) and has since been adopted in a wide variety of applications. In the application considered here, image scans of roots harvested from soil can contain significant levels of noise, as exemplified in Fig. 4A. The noise level is dependent on many factors such as soil type, abundance of root hairs, and the degree of care exercised in root washing and brushing. In such cases, the traditional thinning algorithm can artificially induce false branches at noise points along the boundaries of roots. As a consequence, significant errors could be incurred in the estimation of root number, an important trait used to phenotype plant roots (Kumar *et al.*, 2014). Although there exist enhanced algorithms (Cai, 2012) using oriented filters to calculate oriented energy for thinning, where oriented energy is robust to noise, such improved algorithms cannot guarantee root connectivity and will also result in an overestimation of root numbers. In the approach presented here, a criterion is introduced to determine whether a boundary point should be removed or retained in order to satisfy the condition of connectivity (see Supplementary Fig. S1 at *JXB* online). With this additional constraint it is can confirmed that the proposed program is able to maintain root connectivity while remaining robust to noise.

#### Automatic determination of primary roots using graphics optimization

Accurate analysis of the physical characteristics of primary and lateral roots, respectively, is important for a detailed characterization of a plant’s response to stress and nutrient availability (Drew, 1975). Consequently, a phenotyping characterization tool would need to have the capability of distinguishing primary roots from lateral roots, which, as stated earlier, is one of the innovative features of this procedure. In addition, the RootGraph algorithm is also able to quantify the properties of lateral roots that are specifically associated with or connected to a given primary root. In this way, one can make a more accurate assessment of the ratio of lateral root properties to primary root properties as well as possibly ascribe differences in functionality to specific primary roots. In this section, the novel graphics optimization algorithm employed to distinguish primary roots from lateral roots is described. This ability, incidentally, is particularly useful in the analysis of outcomes of a split-root design experiment (wherein a plant root system is allowed to develop and grow in a container having a vertical, impermeable divider separating different soil treatments (Ruffel *et al.*, 2011; Song *et al.*, 2013).

#### The generation of undirected graph

The first step is to create an undirected graph from the root skeleton obtained using the thinning algorithm. The undirected graph is defined as

G=(V,E) (3)

where *V* is a set of vertices or nodes and *E* is a set of edges or curves. Vertices were used to represent the points of juncture in the root skeleton. A vertex is called an end vertex if it links to one edge only. Edges were used to represent root skeleton curves between vertices with each edge having whole attributes such as length, *l*_*i*_ , and specific score, α_*i*_, as well as local attributes such as thickness as a function of contour length along the edge. The specific score of an edge, α_*i*_, is defined through the formula

$$\alpha_{i}=\left(\bar{T_{i}}-\theta_{t}\right)l_{i}$$ (4)

where *i* is the index of the edge, $\bar{T_{i}}$ is the average thickness of the edge, $\theta_{t}$ is a threshold constant, $\theta_{t}=2\sigma_{1}$, and σ_1_ is the thickness variance of lateral roots. Note that specific score is crucial to the automated identification of primary roots by using optimization algorithms.

#### The reduction of graph complexity

With the graph created in the previous step, the problem of identifying a primary root is equivalent to the problem of finding the optimal path of edges with the maximum of accumulated specific score, $\sum \alpha_{i}$, between any two given vertices. Note that with knowledge of the two terminating points (vertices) of a primary root, the complete contour can be identified using the A* search algorithm (Zeng and Church, 2009) directly to find the shortest path between the two vertices, without reference to any specific path scores, as is done in the case of RootNav (Pound *et al.*, 2013). In such an event, if the graph possessed a tree-like structure, the complexity of finding the primary root from one vertex to the other is of the order of $N_{\nu }^{2}$, where $N_{\nu }$ is the total number of vertices. In the case of a fully automated primary root identification procedure, i.e. one without user identification of end vertices, a primary root’s vertices are unknown and the procedure must determine the optimal path based on specific scores between all possible vertex pairs. In this case, the complexity of automatically finding a primary root is of the order of$N_{\nu }^{4}$.

When *in situ*, a plant’s RSA is a 3D tree-structure, but when laid out on a 2D flatbed scanner as shown in Fig. 4B, the system is more complex from a computational point of view. The ensuing graph can be very complex and each vertex can be associated with multiple edges including loops and forks, etc. In the general case, the complexity of finding the optimal path between two given vertices increases exponentially with number of vertices. Clearly, the automatic identification of primary roots is not viable given the computational cost and memory requirements since a root graph can have more than 100 vertices. Therefore, it is necessary to reduce the complexity to a more manageable level.

To reduce the number of vertices and edges of the graph, the simplest of cases is first considered. Firstly, all loop edges can be removed as they are caused by root hairs and soil. Secondly, a set of rules can be established adherence to which will simplify the problem.

- **[Rule 1]** All edges that both possess negative specific scores and are linked to end vertices can be removed as they represent lateral roots.
- **[Rule 2]** Given any two neighbouring vertices, remove all edges bar the one having maximum specific score.

Usually, the diameters of lateral roots are smaller than those of primary roots for the same plant. However, the diameters of some short lateral roots near the ends of primary roots are similar in size, as shown in Fig. 4B. Their specific scores are therefore positive. An effective way to prune away lateral root edges linked to an end vertex is to compare their specific scores with those of neighbour edges.

- **[Rule 3]** If a root’s specific score is the smallest among neighbour edges, that end vertex is discarded along with the edge linking it to the vertex in question.
- **[Rule 4]** After pruning, remove the remaining vertices having only two linked edges. The above process is repeated until no further pruning can be performed. At this stage, the number of vertices is usually <10 allowing the A* search algorithm to cope with a more manageable complexity of *O*(10^4^). Should there be >10 vertices in a graph, root diameters are used to further prune edges and vertices.
- **[Rule 5]** Analogous to the method employed by SmartRoot (Lobet *et al.*, 2011), the edge with the smallest specific score from a vertex linked to multiple edges (due to root overlap) can be pruned away, under the reasonable assumption that the diameter of a lateral root is smaller than that of the primary root at their junction. This assumption is always upheld in theory, but not always in practice for root images containing two or more overlapping lateral roots, as is visible on the left hand side and bottom right corner of Fig. 4B. Therefore, this pruning rule could potentially break one primary root into two or more pieces and result in an erroneous count of primary roots. Nevertheless, the advantage of this rule is that the A* algorithm can always complete its path search in a reasonable period of time.

#### Root identification and root phenotyping

**Fig. 5.**
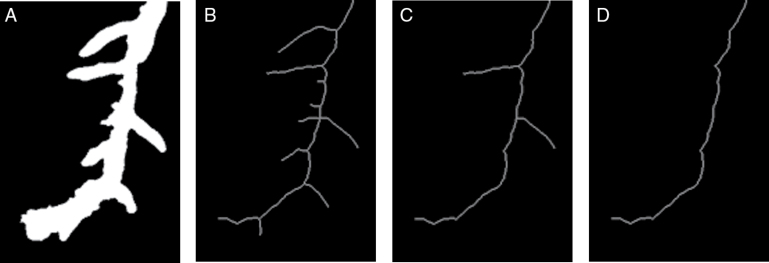
The process of reducing the complexity of root structure for root identification: (A) a segmented root image; (B) the skeleton of (A); (C) the root during the process; and (D) the final root structure for primary root identification.

After pruning, one can comfortably apply the A* algorithm to find the optimal paths between terminating vertices, i.e. those that correspond to primary roots. Following the identification of all primary roots, one can refer back to the original graph to easily calculate the geometric properties of primary roots such as length, local diameter, surface area, and volume. One can then determine the lateral root count for each primary root by counting the number of non-primary root vertices. From the graph, lateral roots can thus be grouped based on their linkage to the primary roots (Fig. 5). The major advantage over SmartRoot and RootNav is that this approach is fully automated and is thus suitable for high-throughput, root analysis.

## Results and discussion

In a recent publication (Kumar *et al.*, 2014) a technique was presented that identified root tips from a large set of corner features (plant related or unrelated) and gave their geometric location in an image. Not only was the number of roots contained in an image of a plant root system quantified, but root tips belonging to primary roots were distinguished from those belonging to lateral roots. As a precursor to a full spatial reconstruction (in either 2D or 3D), the methodology gave an estimate of the number of primary roots and the number of lateral roots contained in that image. The success of the method was measured against manually determined root numbers and the performance was quantitatively compared with those of popular programs EZ-Rhizo (Armengaud *et al.*, 2009) and WinRHIZO^TM^. For the images considered, the method, now referred to as RTipC (for Root Tip Counter), achieved high statistical accuracy compared with a manual count and outperformed both WinRHIZO and EZ-Rhizo.

The RootGraph method described in this paper, quantifies a larger span of root traits than RTipC including total root length, surface area, root volume, average root diameter for primary vs lateral roots, as well as the total number of primary vs lateral roots. Many of these features are also estimated by the WinRHIZO program. However, it has been previously shown that WinRHIZO gives a poor account of lateral and primary root number (Kumar *et al.*, 2014). The new method also has the innovative feature, not previously considered, of being able to identify these same characteristics specifically of the lateral roots emerging from any given primary root. Thus, the method can give a ‘primary root by primary root’ analysis of lateral root features, such as lateral root number per centimetre of primary root. Given the overlap in quantities captured by WinRHIZO and RTipC, this naturally leads one to compare the performances of these methods to RootGraph, as well as evaluate the accuracy of RootGraph against manually determined properties. It is noted that quantitative comparisons with other software alternatives were presented in Kumar *et al.* (2014) as were discussions of qualitative differences. For reasons of space, the remarks and conclusions are not repeated here.

Two systems have been chosen for method evaluation. The systems offer the opportunity to test performance when applied to roots of varying complexity. The first system comprised roots of 14-d-old plants from 10 double haploid lines of barley; all plants were subject to the same growth treatment. Twenty flatbed scanned images were used in this analysis. The second system comprised flatbed scanned images of 6, 9, 13, and 17-d-old roots of the Australian wheat cultivars, Kukri and Gladius. Two treatments were considered, NN and LN treatment as outlined in Methods. Twenty-two images in all were analysed, covering both treatments.

For validation (i.e. assessment of accuracy) only the following traits were considered: total primary root number, total lateral root number, lateral root count per primary root, total length of primary root, and total length of lateral root. Only a sample subset of all available images were used in this validation exercise. For reasons of difficulty, there was no manual determination of geometric properties such as root surface area or root volume.

### Root count

The process begins with the simplest of root traits—primary and lateral root count—traits that can be estimated by all three programs: RTipC, WinRHIZO, and RootGraph. In Table 2, the total root count of primary and secondary roots across the subset of 20 barley images and 18 wheat images are summarized.

**Table 2.** Comparison of results of application of (a) RootGraph, (b) RTipC, and (c) WinRHIZO software, and manual labelling of primary and lateral root numbers extracted from subsets of images of barley (*n*=20) and wheat cultivar Kukri (*n*=18) The columns show primary (Prim), lateral (Lat), and total (Tot) root counts accumulated over all manually labelled images. The inequalities beneath the barley data (only) refer to the relative error in lateral root count for the given method as experienced across the range of images. Negative values refer to underestimated root counts. As WinRHIZO does not explicitly differentiate between primary and secondary roots, a diameter threshold of 0.338mm subjectively applied to differentiate between primary and lateral roots. As WinRHIZO grossly overestimates root numbers generally, no effort was made to categorize the counts in primary and lateral roots.

	Manual			RootGraph			RTipC			WinRHIZO		
	Prim	Lat	Tot	Prim	Lat	Tot	Prim	Lat	Tot	Prim	Lat	Tot
Barley	120	1836	1956	120	1888	2018	120	1745	1865	123	4379	4502
				−7%<Δε(lat)<14%			−22.3%<Δε(lat)<20%			63%<Δε(lat)<211%		
Wheat	90	2097	2187	91	2321	2412	54	5450	5504			10493

As discussed in previous work (Kumar *et al.*, 2014), WinRHIZO does not automatically distinguish between primary and lateral roots as is possible using RTipC and RootGraph. Instead, a user-defined value of root thickness (based on an analysis of root histograms of root length to root thickness to determine the average root thickness, an example of which appears in Fig. 3) as a means of differentiating primary roots from lateral roots. The thickness threshold chosen in this study was 0.338mm.

It is acknowledged that the manually determined values, especially the lateral tip numbers, may have some intrinsic error associated with them, which have not been quantified. Nevertheless, both RootGraph and RTipC estimate the primary root count with 100% accuracy for each image of barley and 99% accuracy for each wheat image. In the case of WinRHIZO, the threshold thickness setting of 0.338mm ensured the least error in the estimation of primary roots. Despite the overall acceptable total error of +2%, there were both undetected and falsely detected primary roots across the set of images, as indicated by the inequality range quoted in Table 2. This variation led to an overall compensation of errors at the level of total root count.

Determination of lateral roots is the more significant discriminator of accuracy and relative performance. Based strictly on the first row results in Table 2, the method introduced here clearly outperforms the other two methods, with an overall error rate of 3% and with an image-to-image variation of error ranging from −7% (undetected roots) to 14% (false detections). This performance is closely followed by RTipC, which demonstrates a similar behaviour to RootGraph in that it failed to identify some actual roots in some images (max error of −22%) and falsely detected roots in other images (max error of 20%) to result in an overall accuracy of 95%. WinRHIZO performs well in terms of primary root counting. However, as demonstrated in Kumar *et al.* (2014), WinRHIZO does not perform well in the quantification of total lateral root number and total root count overall. In this case, the difficulty lies in WinRHIZO’s inability to cope with gradients in the background: the non-negligible noise gradients are registered by WinRHIZO and, as they are well below the thickness threshold, are classified as lateral roots. Conventional thinning algorithms such as the Zhang–Suen method (Zhang and Suen, 1984)—on which WinRHIZO is based—are sensitive to small levels of noise (Liu *et al.*, 2003; Kumar *et al.*, 2014).

With regard to the results for wheat, RootGraph achieved the best performance. This is partially due to the smoothing effect of the thinning algorithm in this approach and partially due to the initial estimate of root parameters, which allows this approach to automatically adopt different image resolutions and to reject some falsely detected root tips. It was noticed, however, that in the case of wheat roots some errors were encountered in the process of separating primary roots from lateral roots. This is due to two contributions. The first contributing factor is broken primary roots, while the second is the violation of [Rule 5] (see Materials and Methods), when the roots are very complex. The second issue can be resolved with the use of a computer with larger memory.

With regard to the poorer performance of RTipC in analysing wheat roots, RTipC is a machine learning-based method for which training was here based on barley root images. It is well known that machine learning approaches can be affected by mismatches between actual data and training data (Jones, 2005). Contributions to mismatching can arise, for example, from different image preparations or different image resolutions (as is likely the case of the present wheat scans). Better accuracy can be achieved by RTipC if root scans are prepared similarly with similar resolution to the images used as training data.

**Fig. 6.**
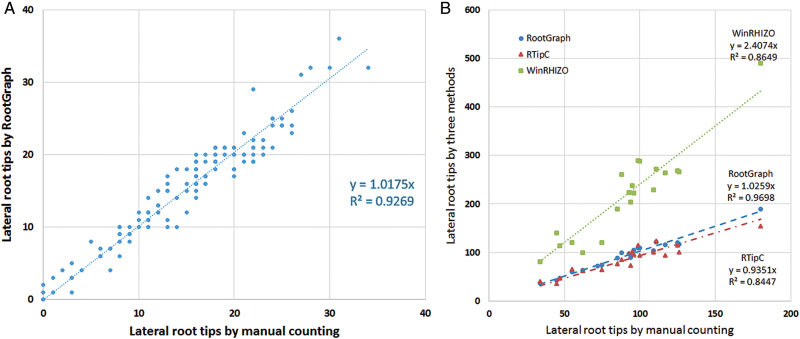
Scatter plots of software determined lateral root counts of barley vs manually determined root counts. (A) RootGraph estimates of lateral roots counts per identified primary root compared with manual benchmarks; and (B) Comparison among RootGraph, RTipC, and WinRHIZO estimates of lateral roots counts per barley plant versus manual data.

Automated root analysis programs such as WinRHIZO and RTipC cannot assign lateral root characteristics to individual primary roots. However, this is now possible with the RootGraph algorithm. In Fig. 6A, a scatter plot is shown of the lateral root count associated with identified primary roots for the barley plant series. The least squares fitting function, y=1.0175x, has a slightly higher slope than the ideal case of *y* = *x*, where *x* is the manual count of lateral root tips per primary root. This difference means the RootGraph algorithm overestimates slightly the lateral root tip count. The *R* ^2^ value of 0.9269 indicates a strong correlation between the two data sets, while still highlighting the high degree of variation in lateral tip count, which is not captured by this simple linear model. Fig. 6B shows the results of RootGraph, RTipC, and WinRHIZO on the lateral root tip count for individual plants. In terms of this phenotypic trait, RootGraph returns the highest *R* ^2^ value and smallest (positive) bias; RTipC also shows a high *R* ^2^ value but tendency to underestimate lateral root counts. WinRHIZO has significantly overestimated the lateral root count, although it returns a surprisingly high *R* ^2^ value, indicating a consistent behaviour.

### Geometric characteristics

Both root length and root surface area are physiologically relevant to root function, in particular to the uptake of water and nutrients. The amount of material (water and nutrients) taken up per unit time is proportional to root surface area and root length (Laperche *et al.*, 2006). This functionality is particularly pertinent to lateral roots. Root volume is a third geometric feature. This is proportional to root biomass, which also has physiological relevance. For example, living cells in root tissues such as the cortex have the capacity to load toxic Na^+^ ions in their vacuoles as a means of defence against salt stress (Apse and Blumwald, 2007). Root volume is thence a measure of the product of cell volume and cell number within a given tissue region. Of these three geometric quantities, only root length has been determined manually. For this aim ImageJ (Schindelin *et al.*, 2012) was used to manually place marker points along a root and to link these to create a curve with which to estimate the length of the root. This process is repeated for all roots to be measured.

**Fig. 7.**
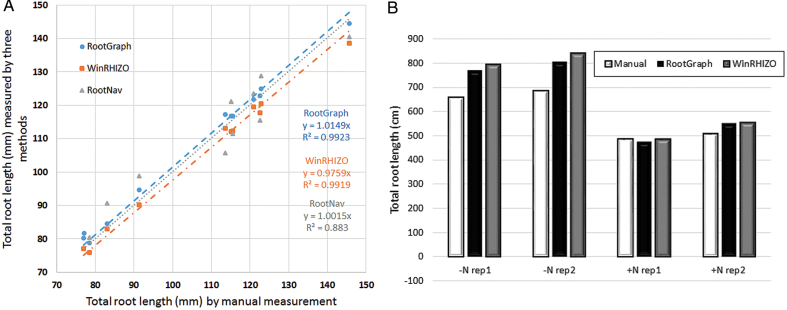
Comparisons between estimates of total root lengths as determined by manual means, RootGraph, WinRHIZO, and RootNav software. (A) Scatter plots of root length calculations using the analysis tools applied to images of barley roots compared with manual measurements. (B) Bar graph comparison between results of RootGraph and WinRHIZO and manual measurements across four selected images of the wheat cultivar Gladius under LN (first two sets of columns) and NN conditions (second set of columns).

To assess the accuracy of both RootGraph and WinRHIZO for the calculation of total (accumulated) root length, a selected number of images were assessed manually. Figure 7 gives a comparison of the two techniques against manual measurements of barley (Fig. 7A) and wheat data (Fig. 7B). In Fig. 7A results of RootNav applied to the barley images are considered to provide a third, independent estimate of root length. This figure shows that all methods perform quite well. RootGraph and RootNav have a negative bias compared with that of WinRHIZO in that both of the former methods underestimate root length. However, RootNav has a relatively lower *R* ^2^ value highlighting an inconsistency due to errors in finding the shortest paths. It is somewhat surprising that WinRHIZO has almost the same the *R* ^2^ value as that of RootGraph, 0.99, despite it overestimating root tip number. On the other hand, it should be remembered that, in contrast to RootGraph, WinRHIZO does not automatically separate primary roots from lateral roots. Moreover, in this comparison, the WinRHIZO threshold was set a priori to advantageously give the least error in WinRHIZO’s primary root count. Without prior knowledge of manually determined root number, it would not be possible to separate this information. It is nevertheless encouraging that WinRHIZO and RootGraph agree on primary and lateral root lengths. Figure 7B also demonstrates that RootGraph performs well when applied to very complex root systems, as in the case of wheat roots grown in nitrogen deficient soil where the total root length is greater than 600cm. It is clear from Fig. 7A, B that root length estimations by all three methods are generally consistent. Nevertheless, RootGraph performs marginally better overall than WinRHIZO. As a final comment, information that is not available but would be of interest to have, is the degree to which the under- and overestimation of individual root lengths, cancel to give the overall marginal error in WinRHIZO’s results.

**Fig. 8.**
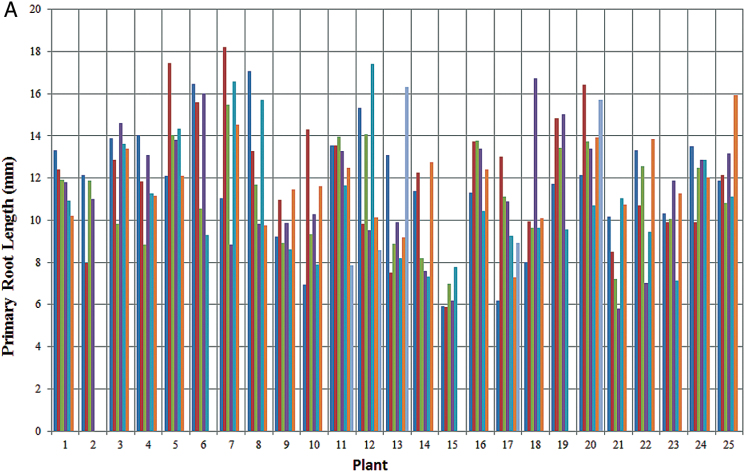

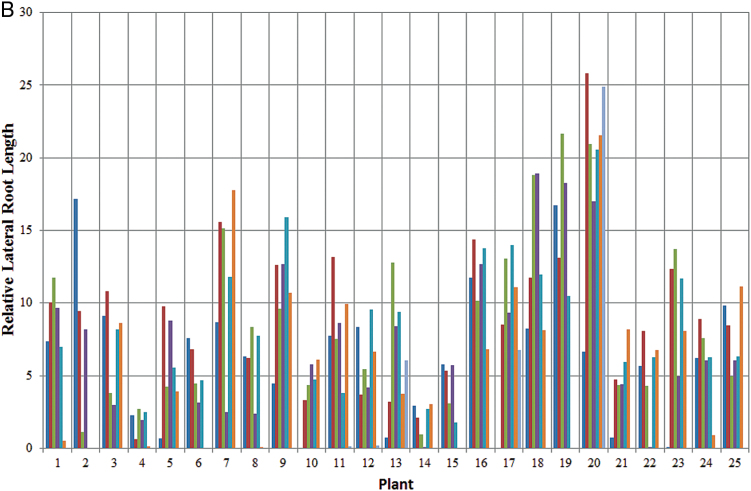
Root lengths from the barley root image series as determined by RootGraph. (A) Length of each identified primary root in a given image in the series. (B) Total length of lateral roots relative to the length of the primary root bearing it, again in the series of 25 barley root images.

With regard to individual root lengths, Fig. 8A, B demonstrate the capability of RootGraph to quantify the lengths of each individual primary root identified in a given scanned image. More significantly. Fig. 8B demonstrates the software’s innovative ability to quantify lateral root features associated with a given primary root. This particular figure features total lateral root length per length of primary root to which the laterals emerge. The quantity shown is effectively an average linear lateral root density, a phenotypic trait that is relevant to a plant’s water and nutrient uptake ability. Although not shown for reasons of space, WinRHIZO performs comparably well with RootGraph in this capability, as already suggested by the results shown in Fig. 7A, B. This is somewhat surprising, given that it vastly overestimates lateral root number (Table 2). The contradiction can be resolved by understanding that the noise features that WinRHIZO attributes to lateral roots have little impact on geometric results. Nevertheless, WinRHIZO’s outputs should be considered cautiously owing to its tendency to falsely classify lateral roots.

Given the inherent difficulty involved, manual measurements of other geometric features such as surface area and volume were not undertaken. Consequently, and in the interest of conserving space, any area or volume analysis using RootGraph has not been presented, even though the program does produce these outputs as a matter of course. However, to summarize, both surface area and volume calculations using RootGraph are based on the assumption that the root adopts the shape of a cylinder locally, with diameter equal to the perpendicular width at that point. If δ*l*(*s*) is the increment in length at a distance *s* from the root tip and *w(s*) is the diameter at *s* measured perpendicular to the root centreline vector, then the increment in surface area and volume are π*w*(*s*)δ*l*(*s*) and $\pi w{\left(s\right)}^{2}\delta l(s)/4$, respectively. Total surface area and total root volume (primary and lateral roots taken separately) are then an accumulation of these increments. Given the higher order nature of the calculations involved in these quantities, one would anticipate greater error than that shown by the individual quantities. For WinRHIZO, it was found that the setting of the segmentation threshold can significantly change the total surface area and volume. This is expected as the width of roots is just a few pixels and surface area and volume can vary significantly if the threshold changes the root width by even one pixel.

## Summary remarks

A fully automated algorithmic tool for the complete quantitative characterization of root systems that have been cut, spread, and flatbed scanned is presented here. This work builds upon a number of generations of numerical approaches aimed at providing quantitative information on root system features of biological relevance. Although it may not be suitable for all types of images, the method presented here improves upon earlier proposals in a number of ways. Generally, the method is fully automated and robust and therefore completely up to the task of high-throughput applications. Secondly, the method represents a major advance by quantifying properties of both embryonic (primary) roots and post-embryonic (lateral) roots, including the possibility of characterizing second, third, and fourth order lateral roots. In this way, associations between properties of lateral roots (such as lateral root density) to their connected primary root may be deduced. This greatly improves upon the value of current coarser-grained relationships established through comparisons of root-system-averaged *lateral* root properties with root-system-averaged *primary* root properties.

In this work the important advance, first presented in a previous paper (Kumar *et al.*, 2014), of distinguishing between primary and lateral roots is maintained. The approach advocated here is more robust in that it utilizes image adaptation and graph optimization and does not rely on any statistical learning. The utility of the method has been demonstrated through an analysis of the complex roots of barley and wheat, with the latter grown under LN and NN conditions. Generally, the method is applicable to analysing roots of dicot or monocot plants grown in soil environments in order to quantify plant responses to nutrient and water stresses. Results have shown that any noise caused by soil particulates accompanying the root extraction process, but remaining after cleansing of roots, can be removed using RootGraph.

Finally, the methodology espoused here can be further developed to assess camera images of fully visible three dimensional root system architecture as opposed to 2D root scans. This will provide a future opportunity to quantify root length, surface area, volume, and other important traits such as 3D root system architecture. A copy of the program is available for download at https://onedrive.live.com/redir?resid=D417979EECAC63C4!2537&authkey=!AHu7kQAVkcwff2c&ithint=folder%2czip and www.plant-image-analysis.org/software/RootGraph.

## Supplementary material

A detailed description of the thinning algorithm is available as Supplementary material at *JXB* online.

Fig. S1. Algorithm schematics.

Supplementary Data
