# Supplementary material for: RootGraph: a graphic optimization tool for automated image analysis of plant roots
Source: J Exp Bot. 2015 Jul 29;66(21):6551–62. doi: 10.1093/jxb/erv359 (PMC4623675; doi:10.1093/jxb/erv359)
Supplement: Supplementary Data [file supp_66_21_6551__index.html]

RootGraph: a graphic optimization tool for automated image analysis of plant roots — RootGraph: a graphic optimization tool for automated image analysis of plant roots — Supplementary Data 

# RootGraph: a graphic optimization tool for automated image analysis of plant roots

## Supplementary Data

Data files

- Supplementary Data - Supplementary Data
