# Supplementary material for: RootGraph: a graphic optimization tool for automated image analysis of plant roots
Source: J Exp Bot. 2015 Jul 29;66(21):6551–62. doi: 10.1093/jxb/erv359 (PMC4623675; doi:10.1093/jxb/erv359)
Supplement: Supplementary Data [file supp_erv359_jexbot153163_file001.pdf]

RootGraph: a graphic optimization tool for automated image analysis of plant roots:

Supplementary Material

JINHAI CAI, ZHANGHUI ZENG, JASON N. CONNOR, CHUN YUAN HUANG, VANESSA MELINO, PANKAJ KUMAR, STANLEY J. MIKLAVCIC

Robust Thinning Algorithm

Thinning (skeletonisation) algorithm is a morphological operation that is used to remove selected foreground pixels from segmented images and to preserve the extent and connectivity of the original regions. Thinning algorithms can be divided into two broad categories, namely iterative and non-iterative. Usually, non-iterative algorithms are faster than iterative algorithms, but they produce less accurate results and are difficult to manipulate. In this paper, we use the Zhang-Suen method (Zhang and Suen, 1984) as the base method, in which it uses the Connectivity Numbers and the Background Numbers in a 3x3 neighbourhood as shown in Figure S1(a) to mark and delete pixels. The Connectivity Numbers $C_n(P)$ and the Background Numbers $B_n(P)$ can be calculated as follows.

$$C_n(P) = \sum_{i=1}^{4}(\overline{n_{2i-1}} - \overline{n_{2i-1}}\,\overline{n_{2i}}\,\overline{n_{2i+1}}) \tag{5}$$

$$B_n(P) = \sum_{i=1}^{8} \overline{n_i} \tag{6}$$

where P is a foreground pixel at the centre of the 3x3 neighbourhood, $\overline{n_i}$ denotes not n_i, n_i=1 if it is a foreground pixel, otherwise it equals 0, and n_9=n_1.

The Zhang-Suen method iteratively removes pixels from contours and preserves the connectivity at the same time. In each iterative process, there are two sub-iterations. In the first sub-iteration, a pixel P can be deleted or set to be a background pixel if the following conditions are satisfied (Liu et al., 2003):

1. $C_n(P)=1$.
2. $2 \le B_n(P) \le 6$.
3. $n_1 n_3 n_7=0$.

4. $n_1 n_7 n_5 = 0$.

In the second sub-iteration the conditions are as follows.

1. $C_n(P)=1$.
2. $2 \le B_n(P) \le 6$.
3. $n_3 n_5 n_7 = 0$.
4. $n_1 n_3 n_5 = 0$.

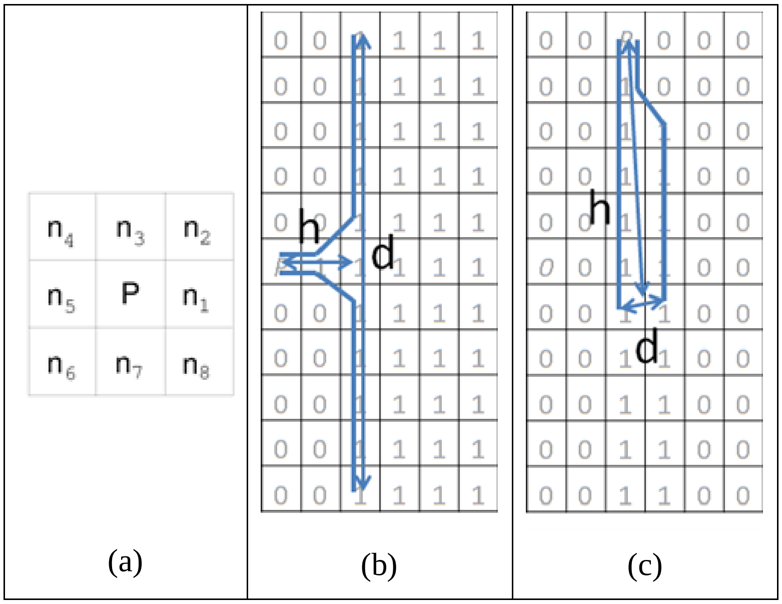

Figure S1. Algorithm schematics: (a) a 3x3 neighbourhood, (b) and (c) the α values of end pixels caused by noise and a true root tip, respectively.

If the pixel *P* is an end pixel, all thinning based on segmented images will keep this pixel as foreground pixel. However, many noisy pixels along contours will become end pixels and further result in false branches in thinning process. In our approach, we use an effective way, which is suitable to the phenotyping of roots grown in soil, to determine whether an end pixel should be deleted (Figure S1(b) & (c)). In this application, there is always some soil present, mainly attached to primary roots after root washing and thus there would be many false branches caused by noisy pixels along contours of primary roots. We refer to two simple cases to demonstrate the idea of the proposed approach. For a given pixel *P*, we trace the contour from both sides of *P* for $\omega/2$ steps. The distance between two tracing ends is *d* and the distance from *P* to the middle of the two ends is *h*. Let α be the ratio between *h* and *d*, so we have $\alpha = h/d$. From the first case, we can find that the value of α is small, the end pixel *P* is likely caused by noise and thus it should be deleted. The

thickness of a short root segment is usually similar, therefore the remainder part of a root segment is thin when the root tip becomes one pixel thick during the thinning process as shown in the second case. In this case, the value of α is quite large. The two simple cases have demonstrated that we can set a suitable threshold (smooth factor) for α to determine whether an end pixel should be deleted. The smooth factor should be selected based on the noise level in root images and its range should be in [0.1, 0.7] regardless of image resolution. Usually, the thicknesses of short noisy branches are much smaller than those of primary roots. Therefore we set ω to the average thickness of primary roots regardless of image resolution, which is low enough to reduce end pixels cause by noise and still able to keep lateral roots.

References

Liu, Z.-C., Cai, J. and Buse, R. (2003). *Handwriting Recognition: Soft Computing and Probabilistic Approaches.* Springer.

Zhang, T. Y. and Suen, C. Y. (1984). A fast parallel algorithm for thinning digital patterns. *Communications of the ACM* **27**, 236–239.
